# Supplementary material for: FERN – a Java framework for stochastic simulation and evaluation of reaction networks
Source: BMC Bioinformatics. 2008 Aug 29;9:356. doi: 10.1186/1471-2105-9-356 (PMC2553347; doi:10.1186/1471-2105-9-356)
Supplement: Additional file 1 — FERN distribution, Version 1.3. This archive contains the FERN source code and binaries as well as documentation and example models in FernML and SBML. [file 1471-2105-9-356-S1.zip › fern/doc/javadoc/fern/network/sbml/package-frame.html]

fern.network.sbml


fern.network.sbml

Classes 
  
MathTree
  
MathTree.ConstLeaf
  
MathTree.GlobalLeaf
  
MathTree.InnerNode
  
MathTree.Node
  
MathTree.VarLeaf
  
SBMLEventHandlerObserver
  
SBMLNetwork
  
SBMLPropensityCalculator
